# Placental Features of Late-Onset Adverse Pregnancy Outcome

**DOI:** 10.1371/journal.pone.0129117

**Published:** 2015-06-29

**Authors:** Lucy E. Higgins, Nicolas Rey de Castro, Naa Addo, Mark Wareing, Susan L. Greenwood, Rebecca L. Jones, Colin P. Sibley, Edward D. Johnstone, Alexander E. P. Heazell

**Affiliations:** 1 Maternal and Fetal Health Research Centre, Institute of Human Development, University of Manchester, Manchester, M13 9WL, United Kingdom; 2 Maternal and Fetal Health Research Centre, St. Mary's Hospital, Central Manchester University Hospitals NHS Foundation Trust, Manchester Academic Health Science Centre, Manchester, M13 9WL, United Kingdom; University of Oxford, UNITED KINGDOM

## Abstract

**Objective:**

Currently, no investigations reliably identify placental dysfunction in late pregnancy. To facilitate the development of such investigations we aimed to identify placental features that differ between normal and adverse outcome in late pregnancy in a group of pregnancies with reduced fetal movement.

**Methods:**

Following third trimester presentation with reduced fetal movement (N = 100), placental structure *ex vivo* was measured. Placental function was then assessed in terms of (i) chorionic plate artery agonist responses and length-tension characteristics using wire myography and (ii) production and release of placentally derived hormones (by quantitative polymerase chain reaction and enzyme linked immunosorbant assay of villous tissue and explant conditioned culture medium).

**Results:**

Placentas from pregnancies ending in adverse outcome (N = 23) were ~25% smaller in weight, volume, length, width and disc area (all p<0.0001) compared with those from normal outcome pregnancies. Villous and trophoblast areas were unchanged, but villous vascularity was reduced (median (interquartile range): adverse outcome 10 (10–12) vessels/mm^2^ vs. normal outcome 13 (12–15), p = 0.002). Adverse outcome pregnancy placental arteries were relatively insensitive to nitric oxide donated by sodium nitroprusside compared to normal outcome pregnancy placental arteries (50% Effective Concentration 30 (19–50) nM vs. 12 (6–24), p = 0.02). Adverse outcome pregnancy placental tissue contained less human chorionic gonadotrophin (20 (11–50) vs. 55 (24–102) mIU/mg, p = 0.007) and human placental lactogen (11 (6–14) vs. 27 (9–50) mg/mg, p = 0.006) and released more soluble fms-like tyrosine kinase-1 (21 (13–29) vs. 5 (2–15) ng/mg, p = 0.01) compared with normal outcome pregnancy placental tissue.

**Conclusion:**

These data provide a description of the placental phenotype of adverse outcome in late pregnancy. Antenatal tests that accurately reflect elements of this phenotype may improve its prediction.

## Introduction

According to the 2010 Europeristat review of perinatal mortality in 31 countries approximately 26% of all stillbirths occurred at or beyond 37 weeks’ gestation, a further 22% occurred between 32–36^+6^ weeks’ gestation [1]. Similar patterns are seen in the United States [2] and New Zealand [3]. Critically, stillbirth rates in high-income countries have shown little improvement in recent decades [4]. Despite the availability of an intervention to prevent late-gestation stillbirth (delivery), current antenatal screening strategies (including sonographic fetal size assessment and umbilical artery Doppler) have not been shown to reduce perinatal mortality in the general obstetric population [5], either because of poor sensitivity or lack of a “test and treat” study protocol, resulting in failure to deliver fetuses with abnormal test results and subsequent fetal demise [6].

A simple and widely employed method to monitor fetal wellbeing is maternal perception of fetal activity [7, 8]. Pregnancies with reduced fetal movement (RFM) have an increased risk of adverse pregnancy outcome, including two- to three-fold increased risk of fetal growth restriction (FGR) and stillbirth [9, 10], with increased incidence of neurodevelopmental problems [11]. RFM is thought to occur as a compensatory response to limited delivery of oxygen and nutrients to the fetus by a dysfunctional placenta [12, 13]. In a systematic review and related Delphi exercise, the International Stillbirth Alliance highlighted RFM as a key priority area for preventing stillbirth in high-income countries [4]. Meta-analysis suggests that although using specific “alarm limits” and formal counting of fetal movement does not decrease perinatal mortality [14], maternal and professional awareness of the importance of fetal activity (and subsequent investigation to identify fetoplacental compromise) is associated with reductions in overall perinatal mortality / adverse pregnancy outcome in study populations [15].

In support of the hypothesis that RFM represents fetal compensation to placental dysfunction, placentas from pregnancies complicated by RFM (hereafter referred to as RFM placentas) display a range of pathologies similar to those observed following stillbirth [16–18] (Table 1) in terms of reduced gross placental size, reduced villous vascularity, increased villous thrombosis and infarction and altered maternal circulating placentally-derived hormone concentrations. However, these studies largely focused on structural, rather than functional, changes and have not distinguished between RFM pregnancies with normal or adverse pregnancy outcome. Warrander et al. demonstrated evidence of reduced amino acid transporter activity in placentas from adverse outcome pregnancies [16], but to date there has been no assessment of placental vascular or endocrine function in RFM. This is important as established clinical investigations to detect placental dysfunction (in terms of Doppler assessments of materno- and fetoplacental blood flow and maternal plasma hormone measurements) focus on these facets of placental physiology [19].

**Table 1:** Placental features associated with stillbirth, fetal growth restriction and reduced fetal movements.

	Stillbirth	FGR	RFM
**Placental Structure**			
**Trimmed placental weight**	↓	↓	↓
**Mean placental diameter**	Unreported	↓	↓
**Placental “roundness”**	Unreported	Unreported	↔
**Central cord insertion**	↓	↓	↓
**Villous vascularity**	↓	↓	↓
**Infarction / thrombosis**	↑	↑	↑
**Fibrin deposition**	↑	↔/↑	Unreported
**Trophoblast area**	↓	↔/↓	↓
**Exchange barrier thickness**	↑	↑	Unreported
**Syncytial nuclear aggregates**	↑	↑	↑
**Placental Function**			
**Fetoplacental weight ratio**	↑	↔/↑	↑
Endocrine profile*	↓/↑*	↓/↑*	↓*
**Amino acid transfer**	N/A	↓	↓
**Lipid transfer**	N/A	↓	Unreported
**Glucose transport**	N/A	↔	Unreported
**Ion transport**	N/A	↓/↑	Unreported
**Metabolism**	Unreported	Altered*	Altered*
**Placental vascular function**	N/A	Altered	Unreported
**Inflammation**	↑	↑	↑
**Proliferation**	↑	↔/↓	↑
**Apoptosis**	Unreported	↑	↔

Collated from studies in pregnancies complicated by stillbirth [20–24], fetal growth restriction (FGR) [25–33] and reduced fetal movements (RFM) [16–18, 34, 35]. Key: ↑ = increased, ↓ = decreased, ↔ = unaltered, N/A = not applicable

* largely inferred from studies of maternal or cord blood or explant conditioned medium.

This study tested the hypothesis that the placental phenotype associated with adverse pregnancy outcome differs from women who have a normal pregnancy outcome after presentation with RFM. We compared the *ex vivo* placental biometry, vascularity, and vascular and endocrine function of RFM pregnancies with and without adverse outcome.

## Materials and Methods

Ethical approval was received from Greater Manchester North West Research Ethics Committee (11/NW/0650) and all work was conducted in accordance with the Declaration of Helsinki 1975 (revised 2013). Women presenting to St. Mary’s Hospital, Manchester, UK with perceived reduction in fetal activity after 28 weeks’ gestation in singleton pregnancies were asked to participate in the study. Pregnancies complicated by pre-pregnancy hypertension or diabetes, or known fetal abnormalities were excluded. Written informed consent to examination of placental tissue after delivery was obtained.

Adverse pregnancy outcome was defined as previously described [36] if any of following criteria were met: stillbirth or neonatal death, small for gestational age (SGA) birth weight (individualised birth weight centile (IBC) <10 derived by Bulk Centile Calculator (UK) 6.7 software, Gestation Network, Birmingham, UK), five minute Apgar score <7, umbilical arterial pH <7.1 or base excess <-10.0 or admission to neonatal intensive care unit within 24 hours of birth. Placentas were collected within one hour of delivery.

Unless otherwise stated, all chemical reagents used were supplied by Sigma-Aldrich (Poole, UK).

### Placental structure

Placentas were trimmed of their extraplacental membranes and umbilical cord, weighed and photographed, chorionic plate facing upward, alongside a scale bar. Their volume was calculated by fluid displacement. Depth was manually measured at the apparent deepest point of the placental body. Using Image ProPlus 6.0 imaging software (Media Cybernetics, Marlow, UK) placental photographs were analysed to establish placental disc area, length (longest diameter), width (longest diameter perpendicular to length) and minimum distance from cord insertion to placental edge (cord distance). Cord distance was divided by placental length to generate the “cord ratio” in order to take into account overall placental size.

Placentas were placed in random orientation on a sampling board and systematically sampled five times (1cm^3^) along a randomly selected plane bisecting the placenta through the umbilical cord insertion. Biopsies were divided in two, and half of each fixed in 10% neutral buffered formalin (18 hours at 4°C) before paraffin embedding and sectioning. Tissue was immunoperoxidase stained using mouse monoclonal antibodies against the trophoblast marker cytokeratin 7 (CK7; 0.9μg/ml; Dako, Ely, UK), and the endothelial cell marker cluster of differentiation-31 (CD31) (0.16μg/ml; Dako) with non-immune mouse immunoglobulin G at corresponding concentration as a negative control and imaged as previously described [16]. Total villous and trophoblast areas, the number of vessels (CD31 positive structures) and the combined vascular luminal area per field of view were quantified as previously described [37]. The mean values for each index across 10 images per tissue section were taken to represent each tissue biopsy, and the median “biopsy” value was taken to represent each placenta.

### Placental function

#### Vascular function

Small (200–500μm) chorionic plate arteries (CPAs) were dissected from the placenta. Arterial segments (N = 8 per placenta) were loaded onto wire myographs (610M Danish Myo Technology A/S, Aarhus, Denmark). Tissue baths contained physiological saline solution [119mmol/L NaCl, 25mmol/L NaHCO3, 4.69mmol/L KCl, 2.4mmol/L MgSO4, 1.6mmol/L CaCl2, 1.18mmol/L KH2PO4, 6.05mmol/L D-glucose, 0.034mmol/L Ethylenediaminetetraacetic acid; pH7.4] bubbled with 5% O2 at 37°C. Resting vessel diameters were measured at 0.9 of L5.1kPa according to the method previously described by Mills et al. [27], adapted from Wareing et al. [38]. Vessel responses to vasoactive agonists and length-tension characteristics were analysed using Myodata 2.01 software (Myonic Software, Aarhus, Denmark).

Vessel responses to vasoactive agonists were assessed according to the protocol described by Mills et al. [27], to construct concentration-response curves to the thromboxane A_2_ mimetic U46619 (10^-10^M – 10^−5.7^M; Calbiochem, EMD Millipore, Billerica, USA) and the nitric oxide donor sodium nitroprusside (SNP: 10^-10^M – 10^-4^M). Constriction responses were expressed as the maximal pressure generated (V_max_; kPa), the concentration of U46619 at which vessel segments achieved 50% of their maximal response (effective concentration, EC_50_; nM) and the area under the concentration-response curve (AUC; arbitrary units). Relaxation responses were similarly expressed as V_max_ (residual constriction percentage, normalised to time control), EC_50_ and AUC. Arterial length-tension characteristics were determined according to the protocol described by Wareing et al. [38]. Passive tension accumulation was quantified by Tau (the time to doubling of tension, expressed as 1/k, where k is the rate constant of the exponential curve) whilst active tension generation was quantified by AUC. Individual vessels’ peak active tension (PAT, mN/mm^2^) values were recorded, along with the diameter at which this was achieved (DiamPAT, % of normalised diameter).

#### Endocrine function

To assess placental endocrine function, the production and release of human chorionic gonadotrophin (hCG), human placental lactogen (hPL), progesterone, placental growth factor (PlGF) and soluble fms-like tyrosine kinase-1 (sFlt-1) were assessed in the remaining placental villous tissue obtained from systematic random sampling (all five samples from each placenta pooled together). These hormones were selected due to previous work demonstrating altered maternal serum levels in antenatal maternal serum in pregnancies complicated by adverse outcome following RFM [34 –36], FGR [39] or stillbirth [20, 40].

Fresh villous tissue was washed in phosphate buffered saline and divided for RNA extraction (treated with RNAsave (Geneflow, Lichfield, UK) at 4°C for 18 hours), tissue hormone content analysis (lysed in 1.5ml distilled water at room temperature for 18 hours) or assessment of hormone release into culture media (CM) in a villous explant culture model as described previously; explants were maintained on Netwell permeable supports (Corning, via Sigma-Aldrich) at the liquid-gas interface in culture media [in 1L: 100ml CMRL-1066 (Gibco, Life Technologies, Paisley, UK), 10% heat-inactivated fetal bovine serum, 100mg L-glutamine, 50IU/ml penicillin, 50μg/ml streptomycin sulphate, 50μg/ml gentamicin, 100μg/ml hydrocortisone, 0.1μg/ml retinoic acid, 1μg/ml insulin] under conditions of 6% O_2_/5% CO_2_ and 37°C for seven days [41].

RNA was extracted from fresh villous tissue using a *mir*Vana miRNA isolation kit followed by removal of genomic DNA using a TURBO DNA-free kit (both Ambion, Austin, Texas, USA). Nucleic acid concentration and contamination were assessed by spectroscopy using the NanoDrop 2000C (Thermo Fisher Scientific, Wilmington, USA). Reverse transcription (RT) was performed in triplicate using an AffinityScript Multi-temperature RT kit (Agilent, Santa Clara, USA) in an MX3000 thermocycler (Stratagene, La Jolla, USA). Real time quantitative polymerase chain reaction (qPCR) was then performed on RT triplicates using primers specific to TATA-box binding protein (S1 Table) and Brilliant III Ultra-fast SYBR QPCR mastermix (Agilent) with annealing at 60°C, followed by dissociation curve analysis. All qPCRs had efficiencies of 85–105%. The cycle threshold (Ct) of each sample was calculated and gene expression calculated as (2^-ΔCt^). Triplicates with a coefficient of variance (CoV) <25% were pooled for further qPCR analysis. Subsequently qPCR was performed according to the same protocol, on pooled triplicates using primers for genes encoding TATA-box binding protein, YWHAZ, RPL13A, human chorionic gonadotrophin (hCG), human placental lactogen (hPL), placental growth factor (PlGF) and soluble fms-like tyrosine kinase-1 (sFlt-1) along with CYP11A1 (the gene encoding CYP450scc, the rate-limiting enzyme of progesterone synthesis [42]) (S1 Table). TATA-box binding protein, YWHAZ and RPL13A expression between adverse and normal pregnancy outcome placentas were compared to ensure they were appropriate reference genes (p>0.05). Relative mRNA expression levels were calculated relative to the geometric mean of all three reference genes (2^-ΔCt^) according to the recommendations of Vandesompele et al. [43].

Hormone concentrations in villous tissue lysate and explant conditioned media (CM) (discarded after the first 24h of culture then collected daily) were quantified in duplicate using commercially available colorimetric enzyme-linked immunosorbant assay (ELISA) kits (S2 Table). The optical density (OD) of the well contents was read by a multiplate reader (BMG labtech, Aylesbury, UK) at a pre-specified wavelength (S2 Table). Sample content was quantified by comparison to a standard curve. The average concentration and CoV of sample duplicates were calculated; those with CoV above 10% were repeated. The protein content of tissue pellets and villous explants were calculated by dissolution in 4ml 0.3M sodium hydroxide and quantification with Bio-Rad Protein Assay (Bio-Rad Laboratories, Hempstead, UK). Lysate and CM hormone concentrations were then normalised to the concentration of a quality control run in every assay, and adjusted to tissue protein content.

### Statistical analysis

Based on previous observations of placental arterial function, trophoblast area and villous vascularity in FGR [27, 44, 45] we calculated that a minimum of seven placentas per group would be required to observe similar magnitude differences in placental structure and function between adverse and normal outcome pregnancies (power 80%, significance p<0.05). As both functional assessment techniques require fresh placental tissue, simultaneous use of placentas for both was not possible. Therefore, based on a conservative expected adverse outcome rate of at least 15% [34] we calculated that 100 participants would be required to provide adequate power to the study; all placentas were examined macroscopically, with subgroup analysis based on the above power calculation for microscopic and functional analyses. Data were presented as median (interquartile range, IQR) or number (percentage) and differences between groups were assessed by Mann-Whitney U Test or Chi Squared test with Yates’ correction as appropriate using Prism 6 for Mac OS X (Graphpad software Inc., San Diego, USA).

## Results

Participants (N = 100) were recruited between January 2012 and May 2014. Twenty-three pregnancies were classified as adverse outcome with the most frequent adverse outcome category being SGA (21, 91.3%), consequently the median IBC is lower in the adverse versus normal outcome pregnancies. No stillbirths or neonatal deaths occurred amongst the study population. Demographic and pregnancy outcome data are shown in Table 2. There were no statistically significant differences in maternal demographics, features of the RFM episode(s) or mode of delivery between pregnancies ending in adverse outcome compared to those with normal outcome. Structure and function analyses were unaffected by mode of delivery (p>0.05, data not shown). The median time from RFM to delivery was 5 (2–12) days.

**Table 2:** Baseline and delivery characteristics of study participants and their offspring.

	NPO	APO	p
N	77	23	
**Maternal Characteristics**			
Age (years)	29.4 (25.1–32.7)	28.0 (23.0–33.3)	0.50
White European	54 (70.1)	15 (65.2)	0.65
Asian	11 (14.3)	4 (17.4)	0.001
Black	7 (9.1)	0 (0)	0.30
Other Ethnicity	5 (6.5)	4 (17.4)	0.23
BMI (kg/m^2^)	26.3 (23.2–30.3)	25.2 (23.3–32.1)	0.97
Parity	0 (0–1)	0 (0–1)	0.38
Smoker	9 (11.6)	5 (21.7)	0.22
**RFM Characteristics**			
Episode number	1 (1–2)	1 (1–2)	0.93
Episode duration (hours)	36 (12–72)	60 (27–108)	0.13
Gestation at presentation (weeks^+days^)	38^+6^ (36^+1^–40^+4^)	38^+4^ (37^+0^–39^+1^)	0.27
**Delivery Characteristics**			
Gestation (weeks^+days^)	40^+1^ (38^+4^–41^+1^)	39^+1^ (38^+0^–40^+4^)	0.076
Induction of labour	48 (62.3)	14 (60.9)	0.90
Laboured	60 (77.9)	15 (65.2)	0.35
Caesarean	15 (19.5)	7 (30.4)	0.27
Male infant	40 (51.9)	12 (52.2)	1.00
**Outcome Characteristics**			
Live birth*	77 (100)	23 (100)	1.00
IBC	41.7 (23.1–67.2)	5.8 (3.0–9.0)	<0.0001
IBC<10*	0 (0)	21 (91.3)	<0.0001
Apgar score <7 at 5 minutes*	0 (0)	0 (0)	1.00
Umbilical Arterial pH<7.10*	0 (0)	1 (4.3)	0.066
Umbilical Base Excess<-10*	0 (0)	1 (4.3)	0.066
NICU Admission*	0 (0)	1 (4.3)	0.066

Normal outcome (NPO) and adverse outcome (APO) pregnancies differ by outcome characteristics alone. Key: BMI = Body Mass Index, IBC = Individualised Birth weight Centile, NICU = Neonatal intensive care unit. Data are presented as median (interquartile range) or number (%) and are compared by Mann Whitney U Test or Chi squared test (with Yates’ Correction as required).

* denotes a variable included in the definition of APO.

### Placental structure

**Fig 1:**
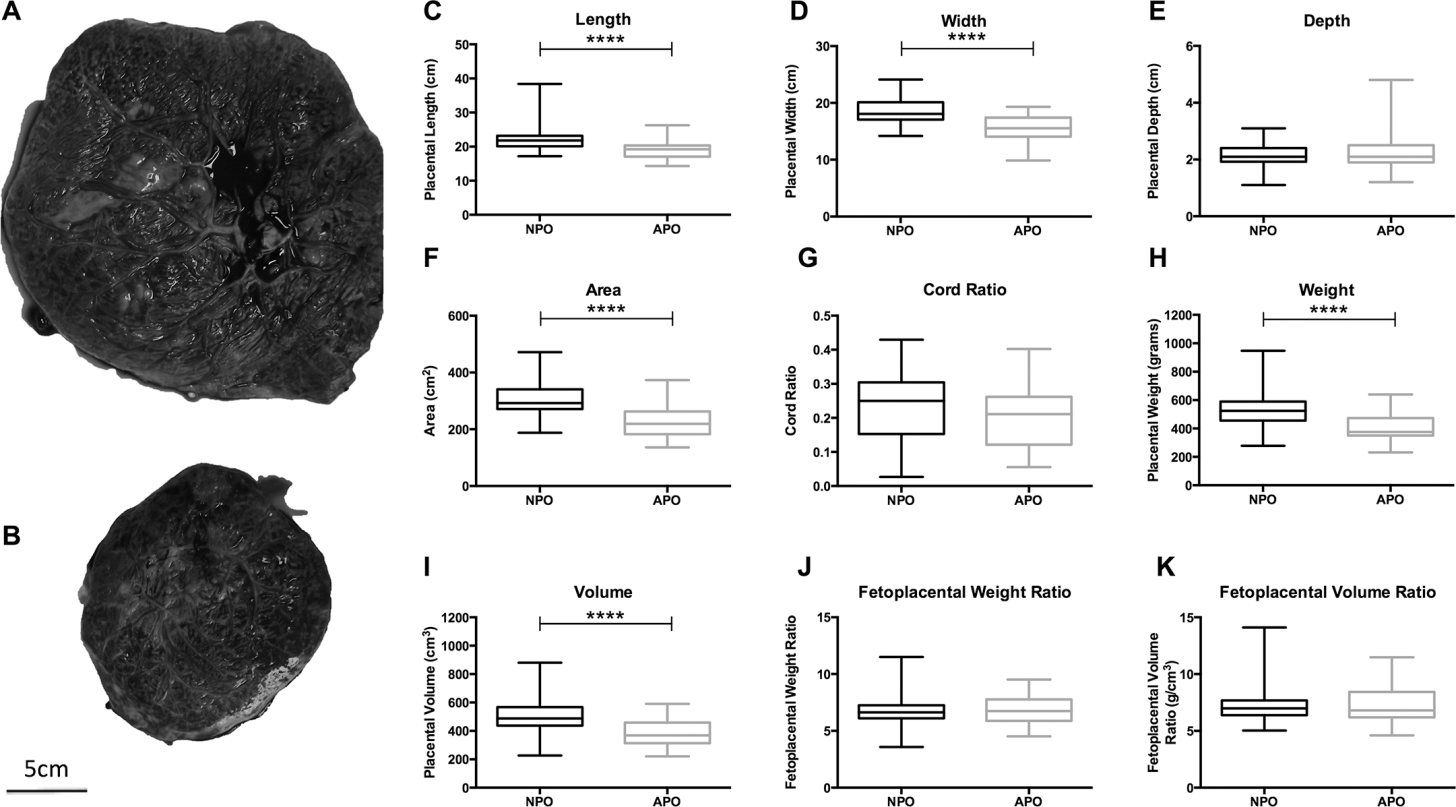
*Ex vivo* placental macrostructure. *Ex vivo* placentas from reduced fetal movement pregnancies with adverse (APO; N = 23) and normal (NPO; N = 77) outcome are biometrically different. Example placental photographs from NPO (A) and APO (B) pregnancies are shown at equal magnification (scale bar = 5cm); box and whisker plots of placental length (C), width (D), depth (E), area (F), cord ratio (G), weight (H), volume (I), fetoplacental weight ratio (J) and fetoplacental volume ratio (K). Data are presented as median (horizontal line), interquartile range (box limits) and range (vertical line) and compared by Mann Whitney U Test. **** denotes NPO vs. APO p<0.0001.

**Fig 2:**
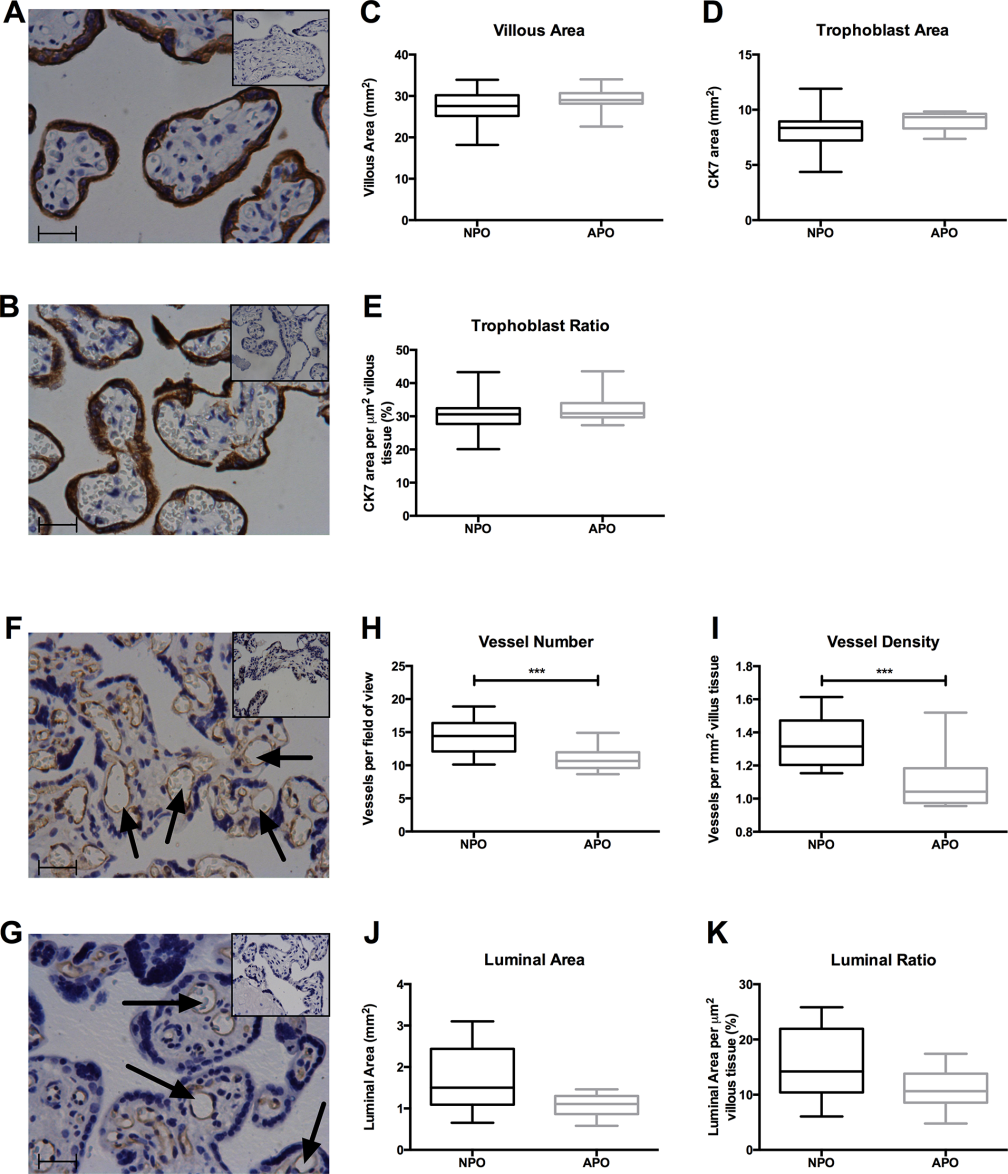
*Ex vivo* placental microstructure. Example images of anti-cytokeratin 7 immunostained villous tissue from normal pregnancy outcome (NPO; N = 20) and adverse pregnancy outcome (APO; N = 9) placentas are shown in (A) and (B) respectively; box and whisker plots of villous area (C), trophoblast area (D) or their relative proportions (trophoblast ratio) (E). Example images of ant-CD31 immunostained villous tissue from NPO (N = 23) and APO (N = 11) placentas are shown in (F) and (G) with vessels identified by arrows; box and whisker plots of vessel number (H), density (I), luminal area (J) and luminal ratio (K). Images are captured at 40x original magnification with scale bar representing 200μm. The corresponding negative control image is inset in the top right corner. Data are presented as median (horizontal line), interquartile range (box limits) and range (vertical line) and compared by Mann Whitney U Test. *** denotes NPO v. APO p<0.001.

Placentas from RFM adverse outcome pregnancies (N = 23) were significantly smaller in terms of length, width, volume, weight and area compared with their normal outcome counterparts’ placentas (N = 77) (Fig 1). There was no difference in placental depth (Fig 1E), the degree of cord eccentricity as measured by the cord ratio (Fig 1G) or the fetoplacental weight or volume ratios (Fig 1J and 1K). When compared to placental centile curves [46] 21/23 (91.3%) and 15/23 (65.2%) of adverse pregnancy outcome placentas weighed below the 10^th^ and 3^rd^ centiles respectively, compared with 36/77 (46.8%) and 17/77 (22.1%) of normal pregnancy outcome placentas (p≤0.0002). Villous area and trophoblast areas were not significantly different between adverse (N = 11) and normal pregnancy outcome (N = 23) placentas (Fig 2A–2F). A reduced number and density of fetal vessels was observed in villous tissue of adverse (N = 9) vs. normal pregnancy outcome (N = 20) placentas (p≤0.002); there was a further trend towards reduced luminal area and ratio in adverse pregnancy outcome placentas (both p = 0.06) (Fig 2G–2I).

### Placental vascular function

**Fig 3:**
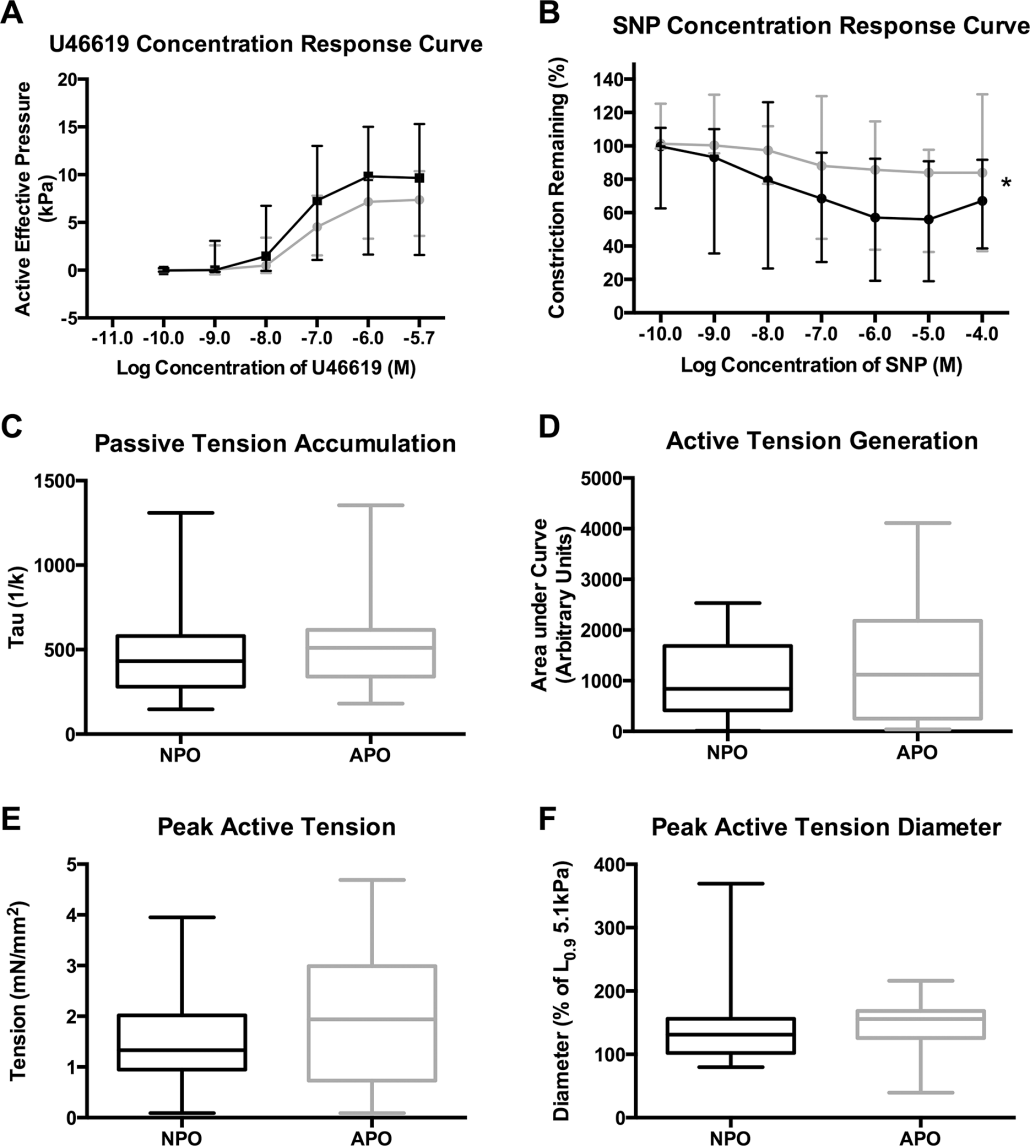
*Ex vivo* placental arterial function. Chorionic plate arteries from adverse pregnancy outcome (APO; N = 8 in grey) placentas show altered responses to vasoactive agonists but length-tension characteristics are unchanged compared to normal pregnancy outcome (NPO; N = 15 in black) counterparts; thromboxane mimetic, U46619 (A) and sodium nitroprusside (SNP) (B) concentration response curves, passive tension accumulation (C), active tension generation (D), peak active tension (E), and peak active tension diameter (F). Data are presented as median and IQR (A&B) or median (horizontal line), interquartile range (box limits) and range (vertical line) (C-F) and all data are compared by Mann Whitney U Test. * denotes NPO vs. APO p<0.05.

In the subset of placentas used to evaluate vascular function, the only statistically significant difference in pregnancy characteristics was that a higher proportion of adverse pregnancy outcome placentas came from obese mothers (adverse pregnancy outcome 5/9 (55.6%) vs. normal pregnancy outcome 1/20 (5.0%), p = 0.002). Eight (88.9%) adverse outcome pregnancies examined for *ex vivo* placental arterial function were SGA, of whom four had an IBC<5. CPA resting diameters were well matched between adverse and normal pregnancy outcome placentas (Adverse pregnancy outcome 365 (240–436) vs. normal pregnancy outcome 288 (190–411) μm). Constriction to U46619 by CPAs from adverse pregnancy outcome placentas was not significantly different from those from normal pregnancy outcome placentas (Fig 3A): AUC (adverse pregnancy outcome 10.3 (7.2–15.9) vs. normal pregnancy outcome 17.3 (12.0–22.6)), V_max_ (7 (5–9) vs. 10 (7–13), p = 0.063), and EC_50_ (49 (33–90) vs. 46 (32–66)). However, CPAs from adverse pregnancy outcome placentas demonstrated impaired relaxation to nitric oxide donation by SNP in terms of AUC (adverse pregnancy outcome 543 (457–595) vs. normal pregnancy outcome 441 (310–530), p = 0.024), V_max_ (87 (57–98) vs. 54 (37–76) %, p = 0.047) and EC_50_ (30 (19–50) vs. 12 (6–24) nM, p = 0.02) compared with those from normal pregnancy outcome placentas (Fig 3B). Length-tension characteristics of the CPAs were similar in adverse and normal pregnancy outcome placentas (Fig 3C–3F).

### Placental endocrine function

All three reference genes were stably expressed between normal and adverse pregnancy outcome placental tissue (p≥0.82). No differences were observed in the relative rates of mRNA transcription for the five genes of interest between adverse (N = 14) and normal pregnancy outcome (N = 21) placentas (Table 3). However, fresh placental tissue from adverse pregnancy outcome pregnancies contained less hCG and hPL protein, and in culture, released less hCG and more s-Flt-1 into CM compared with placental tissue from normal pregnancy outcome pregnancies (Table 4). There were no differences between adverse and normal pregnancy outcome in the other hormones assessed.

**Table 3:** *Ex vivo* placental endocrine function; Relative mRNA expression.

Gene	NPO	APO	p
**N**	21	14	
hCG	0.6 (0.2–26.9)	9.1 (1.0–24.2)	0.15
hPL	163.4 (16.1–911.2)	275.9 (37.6–656.1)	0.62
CYP11A1	2.3 (0.6–6.2)	2.3 (0.8–9.2)	0.55
PlGF	4.0 (1.3–31.4)	7.3 (3.7–19.5)	0.45
sFlt-1	1.4 (0.6–6.4)	2.0 (0.5–7.0)	0.78

Relative gene transcription (compared to expression of TATA-box Binding Protein) of key placental hormones in placentas of normal outcome (NPO) and adverse outcome (APO) reduced fetal movement pregnancies. Key: hCG = human chorionic gonadotrophin, hPL = human placental lactogen, CYP11A1 = gene encoding CYP450scc (key synthetic hormone of progesterone), PlGF = placental growth factor, sFlt-1 = soluble fms-like tyrosine kinase. Values are presented as median (IQR) and compared by Mann Whitney U Test

**Table 4:** *Ex vivo* placental endocrine function; Tissue protein content and release.

Hormone	Lysate protein content			Explant-Conditioned Medium protein content		
	NPO	APO	p	NPO	APO	p
**N**	28	14		15	6	
hCG (mIU/mg)	**55.1 (23.8–102.0)**	**19.9 (11.0–50.2)**	**0.0067**	**160.7 (119.6–295.2)**	**84.6 (69.4–179.1)**	**0.028**
hPL (mg/mg)	**27.0 (9.1–49.8)**	**10.7 (5.9–14.1)**	**0.0062**	89.5 (67.5–103.6)	76.6 (54.0–95.9)	0.40
Progesterone (ng/mg)	426.9 (323.8–605.2)	429.4 (365.2–620.6)	0.88	425.1 (314.5–555.3)	309.9 (179.1–523.3)	0.17
PlGF (pg/mg)	277.3 (89.3–501.8)	133.1 (87.7–308.2)	0.22	342.6 (287.4–480.6)	329.1 (205.3–727.1)	0.59
sFlt-1 (pg/mg)	673.4 (348.6–1230)	812.6 (496.4–2016)	0.36	**5473 (1650–14814)**	**20498 (12978–28914)**	**0.013**

Tissue content and release of key placental hormones in placentas of normal outcome (NPO) and adverse outcome (APO) reduced fetal movement pregnancies. Key: hCG = human chorionic gonadotrophin, hPL = human placental lactogen, CYP11A1 = gene encoding CYP450scc (key synthetic hormone of progesterone), PlGF = placental growth factor, sFlt-1 = soluble fms-like tyrosine kinase. Values are presented as median (IQR) and compared by Mann Whitney U Test

## Discussion

The data reported here support our hypothesis that there is an altered placental phenotype in pregnancies reporting RFM when there is an adverse pregnancy outcome versus those with RFM and a normal pregnancy outcome. In pregnancies complicated by RFM after 28 weeks gestation, placentas from pregnancies ending in adverse outcome are lighter, smaller and less vascularised and also display aberrant vascular and endocrine function compared to normal outcome RFM pregnancies. These structural and functional changes might reduce transplacental transfer of nutrients and oxygen to the fetus, impairing the ability of the placenta to support the optimal growth and development of the fetus, thereby putting the infant at increased risk of perinatal mortality and morbidity. Alternatively, they may confound the relationship between an alternative causal pathology and adverse pregnancy outcome. In any case, such changes merit exploration as they could form the basis of future tests of placental health to assist the identification of the “at-risk” fetus, for example by measurement of placental size and vascularity by placental ultrasound, or examination of placental endocrine function by assessment of placental hormone concentrations in the maternal circulation.

The principal reason for pregnancies in this study to be classified as adverse pregnancy outcome was being SGA at birth (21% of the total study population); this is in excess of the rate in unselected populations (~11% [47]) and in keeping with a previously described increased risk of SGA in pregnancies complicated by RFM [9]. This supports the choice of RFM pregnancies as a population at increased risk of adverse outcome in late pregnancy. The absence of stillbirth in a pregnancy cohort of this size is unsurprising, given the background rate of 5.2 per 1,000 UK births during the time of this study [48]. Given the preponderance of SGA babies amongst the adverse pregnancy outcome cohort, the observed macro- and micro-structural phenotype in these pregnancies is not unexpected (see Table 1), with the exception of the lack of reduction in villous area, which contrasts with that reported by Daayana et al. [44] and lack of significant increase in fetoplacental weight ratios [24]. Nevertheless, our observations provide further evidence that the pathophysiological processes underlying adverse outcome in RFM pregnancies are similar to those observed in FGR and stillbirth. Our findings suggest that the differences in placental structure seen between RFM and healthy controls [16, 17] are most pronounced in pregnancies associated with adverse outcome.

Conversely, the vascular function profile observed in adverse pregnancy outcome placentas contrasts with that described in studies of FGR (commonly defined for research purposes as IBC <5) which demonstrated an increased sensitivity to U46619 [27] and alteration of length-tension characteristics [28]. Possible factors contributing to this difference may be the more physiological oxygen concentrations used in the current study [38], as hyperoxia increases placental arterial constriction [49], differences in maternal BMI and differences in gestational age of samples. The majority of mothers of adverse outcome pregnancies in this study had a BMI>30 kg/m^2^; these women are a subpopulation with an increased risk of stillbirth [50]. Interestingly, obesity is associated with the same insensitivity to nitric oxide donation observed in the CPAs of adverse pregnancy outcome placentas examined in this study [51]. Separate mechanistic studies are required to establish the reasons underlying this observation, with the likely site of abnormality being in the vascular smooth muscle as SNP is an endothelial independent vasodilator [52]. With regard to the effects of gestation, Mills et al. largely used early-onset, FGR requiring preterm delivery which contrasts with our population of predominantly late-onset FGR/placental dysfunction in pregnancies delivering at or around term [27, 28]. This is consistent with observations of altered pathophysiology between early- and late-onset FGR [53]. Critically, late-onset FGR/placental dysfunction is more reflective of the clinical picture that results in stillbirth in late pregnancy when abnormal umbilical artery Doppler waveforms are uncommon [54]. Thus, these pathophysiological changes to placental vasculature in late pregnancy may not be reflected in the umbilical artery Doppler waveform. Further research is necessary to determine which clinical investigations best relate to these abnormalities of placental vessels.

Disruption of placental endocrine function was evident by altered production and release of several placental hormones in adverse pregnancy outcome placentas. Reduced hPL protein content per unit of placental tissue described in this study mirrors the reduction in maternal serum hPL concentrations in adverse outcome pregnancies [34, 55–60] and the down regulation of hPL transcription in placental tissue from SGA infants [61], although we were not able to confirm the latter finding in our study. Similarly, the reduced placental tissue hCG content and release mirrors the association of increased adverse pregnancy outcome risk with low first trimester maternal serum hCG concentration [20], but contrasts with previous reports of enhanced hCG release from placental explants and primary cytotrophoblasts of FGR infants [62, 63]. These reductions in hPL and hCG lysate content are not related to altered trophoblast area or transcription rate. Thus, they may reflect reduced protein synthesis, consistent with post-transcriptional regulation for example by miRNA [64, 65] or post-translational regulation such as the unfolded-protein response secondary to placental stress in FGR [66]. Further studies are required to establish which post-transcriptional regulation mechanisms are involved in these changes in APO placental tissue. Lack of corresponding reduction in lysate PlGF, sFlt-1 or progesterone content suggests that there may be differential effects on net production of individual hormones rather than a global reduction in hormone production or release in adverse outcome RFM pregnancies. Increased release of sFlt-1 during villous explant culture of adverse pregnancy outcome placentas is in keeping with the findings of Nagamatsu et al. [67], Nevo et al. [68] and Gu et al. [69] who observed that release of sFlt-1 is enhanced under hypoxic culture conditions. Collectively, these data (normalised to the amount of placental tissue) do suggest the presence of placental dysfunction rather than simply being an effect of reduced placental size.

The major strengths of our study are the detailed, matched structural and functional characterisation of placental samples with short delivery to collection interval to reduce storage artefact [70]. Additionally, given the predominant growth and vascular development of the placenta in the first half of pregnancy [71, 72], the short interval between presentation with RFM and delivery suggests that this placental phenotype was present at the time of presentation with RFM. Finally, the analyses of placental structure and function were restricted to those with the availability of potential non-invasive methods of *in utero* assessment (for example placental ultrasound, placental arterial circulation Doppler and maternal blood tests). Technologies under development, such as magnetic resonance imaging may in the future allow assessment of placental oxygen and nutrient transfer or metabolism [73–75] which could exploit findings of reduced amino acid transport in adverse pregnancy outcome placentas [16], but these were not considered in this study. Thus, the placental phenotype of adverse pregnancy outcome described here has potential to be translated into clinical practice with minimal safety and acceptability concerns.

The current study has a number of limitations. Outcomes were defined on the basis of a composite primarily influenced by birth weight (the end result of intrauterine growth) and not growth itself, although each component of the adverse pregnancy outcome definition is related to increased perinatal mortality [76–78] and is in keeping with that used by other obstetric studies [34, 36, 79–82]. The limitation of such an outcome definition is reflected in the high proportion of placental weights <10^th^ centile even amongst “normal” outcome RFM pregnancies in the cohort (46.8%). Secondly, PlGF and sFlt-1 were measured separately using commercially available ELISA kits, and not by bedside measurement techniques (Triage by Alere Inc., Waltham, USA and Elecsys by Roche Diagnostics Ltd, Burgess, UK) that might more readily be employed in clinical practice. Although there is a good correlation between PlGF measurements by these techniques [83], it cannot be assumed that the findings of this study are translatable to other measurement methods. Regarding vascular functional assessment, the technique of wire myography isolates the vessels from endocrine and paracrine factors from blood and from the support of surrounding tissue that may influence the *in vivo* vascular phenotype; whole placental cotyledon perfusion studies may assist this in the future. Finally, the external validity of these placental findings, outside the context of RFM, may be questioned. However, when compared to Table 1, the pattern of placental findings described in adverse pregnancy outcome RFM placentas in this study is similar to that of stillbirth and FGR in general, with or without preceding RFM. Thus while the findings would need to be corroborated in other populations and at-risk groups, we believe them to be generally applicable.

A previous Cochrane review found insufficient evidence to recommend or refute routine measurement of either biochemical [84] or vascular placental markers [10]. For such tests to demonstrate significant benefit in preventing stillbirth a standardised combined “test and treatment” intervention is required. The Reduced Movements Intervention Trial (ReMIT) pilot study shows that studies of this nature are feasible [36]. In late pregnancy a treatment (delivery) to prevent stillbirth is available; what is required now to make progress is an accurate test. This study highlights areas of placental health that could form the basis for the development of such tests, for example by measurement of placental size using ultrasound, placental vascularity and vascular function using Doppler ultrasound and placental endocrine function using ELISA of maternal blood. However before such tests are implemented it is important to ascertain whether these aspects of placental structure and function can be reliably assessed *in utero* during late pregnancy with enough accuracy to detect subtle changes in placental physiology and anatomy. This remains outwith the remit of the current study. Only once reliable, sensitive tests of placental structure and function are developed can we hope to significantly reduce placentally related stillbirth rates.

## Supporting Information

S1 Table Primers used to study placental transcription of key placental hormones. Key: ^e^ = Eurofins Genomics, Ebersberg, Germany. ^I^ = Invitrogen, Paisley, UK. TBP = TATA-box binding protein (a placental housekeeping gene), hCG = human chorionic Gonadotrophin, hPL = human placental lactogen, CYP11A1 = gene encoding CYP450scc (key synthetic enzyme of progesterone), PlGF = placental growth factor, sFlt-1 = soluble fms-like tyrosine kinase-1.
(DOCX)

S2 Table Enzyme-Linked Immunosorbant Assay kits used to quantify hormone content of tissue lysate and explant-conditioned media. Key: hCG = human chorionic gonadotrophin, hPL = human placental lactogen, PlGF = placental growth factor, sFlt-1 = soluble fms-like tyrosine kinase-1, DRG = DRG International, Springfield, USA, R&D = R&D Systems, Abingdon, UK, CoV = coefficient of variance.
(DOCX)
