# Supplementary material for: Placental Features of Late-Onset Adverse Pregnancy Outcome
Source: PLoS One. 2015 Jun 29;10(6):e0129117. doi: 10.1371/journal.pone.0129117 (PMC4488264; doi:10.1371/journal.pone.0129117)
Supplement: S1 Table — Key: e = Eurofins Genomics, Ebersberg, Germany. I = Invitrogen, Paisley, UK. TBP = TATA-box binding protein (a placental housekeeping gene), hCG = human chorionic Gonadotrophin, hPL = human placental lactogen, CYP11A1 = gene encoding CYP450scc (key synthetic enzyme of progesterone), PlGF = placental growth factor, sFlt-1 = soluble fms-like tyrosine kinase-1. (DOCX) [file pone.0129117.s001.docx]

SUPPLEMENTARY TABLE S1: Primers used to study placental transcription of key placental hormones.

| **Gene** | **Primer Sequence**  **(5’ – 3’)** | **Accession Number** |
| --- | --- | --- |
| **TBP^i^** | F: CACGAACCACGGCACTGATT  R: TGCAGCACGCGGGTCATGGT | NM_001172085 |
| **YWHAZ^i^** | F: CCTGCATGAAGTCTGTACTGAG  R:TTGAGACGACCCTCCAAGATG | NM_003406 |
| **RPL13A^i^** | F:CGAGGTTGGCTGGAAGTACC  R:CTTCTCGGCCTGTTTCCGTAG | BC0702223 |
| **hCG^i^** | F: TCACTTCACCGTGGTCTCCG  R: TGCAGCACGCGGGTCATGGT | NM_000737 |
| **hPL^e^** | F: TCCTCAGGAGTATGT  R: CACAGCTACCCTCTA | NM_020991 |
| **CYP11A1^e^** | F: TCCAGAAGTATGGCCCGATT  R: CATCTTCAGGGTCGATGACATAAA | NM_000781 |
| **PlGF^i^** | F: GAACGGCTCGTCAGAGGTG  R: ACAGTGCAGATTCTCATCGCC | NM_001207012 |
| **sFlt-1^i^** | F: GGGAAGAAATCCTCCAGAAGAAGA  R: GAGATCCGAGAGAAAACAGCCTTT | NM_001159920 |

Key: ^e^ = Eurofins Genomics, Ebersberg, Germany. ^I^ = Invitrogen, Paisley, UK. TBP = TATA-box binding protein (a placental housekeeping gene), hCG = human chorionic Gonadotrophin, hPL = human placental lactogen, CYP11A1 = gene encoding CYP450scc (key synthetic enzyme of progesterone), PlGF = placental growth factor, sFlt-1 = soluble fms-like tyrosine kinase-1.
