# Supplementary material for: Placental Features of Late-Onset Adverse Pregnancy Outcome
Source: PLoS One. 2015 Jun 29;10(6):e0129117. doi: 10.1371/journal.pone.0129117 (PMC4488264; doi:10.1371/journal.pone.0129117)
Supplement: S2 Table — Key: hCG = human chorionic gonadotrophin, hPL = human placental lactogen, PlGF = placental growth factor, sFlt-1 = soluble fms-like tyrosine kinase-1, DRG = DRG International, Springfield, USA, R&D = R&D Systems, Abingdon, UK, CoV = coefficient of variance. (DOCX) [file pone.0129117.s002.docx]

SUPPLEMENTARY TABLE S1: Enzyme-Linked Immunosorbant Assay kits used to quantify hormone content of tissue lysate and explant-conditioned media.

| **Hormone** | **Company** | **Product ID** | **Optical Density**  **(nM)** | **Range of Detection** | **Intra-assay CoV** |
| --- | --- | --- | --- | --- | --- |
| **hCG** | DRG | EIA 1469 | 450 | 5-1000mIU/ml | 5.5% |
| **hPL** | DRG | EIA 1283 | 450 | 0-20mg/L | 5.2% |
| **Progesterone** | DRG | EIA 1561 | 450 | 0-40ng/ml | 2.6% |
| **PlGF** | R&D | DPG00 | 540 | 0 – 1000pg/ml | 5.0% |
| **sFlt-1** | R&D | DVR100B | 540 | 0-2000pg/ml | 4.8% |

Key: hCG = human chorionic gonadotrophin, hPL = human placental lactogen, PlGF = placental growth factor, sFlt-1 = soluble fms-like tyrosine kinase-1, DRG = DRG International, Springfield, USA, R&D = R&D Systems, Abingdon, UK, CoV = coefficient of variance.
